# Diet Diversity Through the Life-Course as an Opportunity Toward Food Allergy Prevention

**DOI:** 10.3389/falgy.2021.711945

**Published:** 2021-09-24

**Authors:** Giulia C. I. Spolidoro, Domenico Azzolino, Matteo Cesari, Carlo Agostoni

**Affiliations:** ^1^Department of Clinical and Community Sciences, University of Milan, Milan, Italy; ^2^Geriatric Unit, IRCCS Istituti Clinici Scientifici Maugeri, Milan, Italy; ^3^Pediatric Intermediate Care Unit, Fondazione IRCCS Ca' Granda Ospedale Maggiore Policlinico, Milan, Italy

**Keywords:** allergy, tolerance, life-course, pediatrics, older people, diet, gut microbiota, malnutrition

## Abstract

The prevalence of food allergies (FA) is increasing worldwide. Generally, the onset of allergies, including FA, begins in early childhood and may persist and/or develop through the life-course. Even though epidemiological studies have focused mainly on children, allergies can also occur for the first-time during adulthood. Within the prolongation of life, it is expected that allergies will be encountered more often even in older people. Recent findings suggest that an early exposure to diverse food antigens may promote the development of immune tolerance. Accordingly, diet diversity during the first year of life or even earlier may have a positive impact on the prevention of allergies. The anti-inflammatory properties of some dietary nutrients may positively contribute to a tolerogenic immune environment too. Diet diversity is associated with a more favorable microbiome, and increasing evidence suggests a promising role of gut microbiota manipulation in inducing immune tolerance. Unjustified avoidance of allergenic foods may expose to intakes of some nutrients below recommended levels through the life-course, even more in cases of self-diagnosis and treatment of presumed forms of food intolerance. Nutritional strategies including the early exposure to a variety of food antigens are a promising area of research for preventive purposes through the life-course possibly extending positive outcomes to older stages. The aim of this paper is to highlight the role of diet diversity in preventing the development of FA starting in early life, as well as to provide an overview of the main strategies to prevent related nutritional issues throughout the life-course.

## Introduction

Over the last decades, the presence of allergies such as food allergies (FA), asthma, atopic dermatitis (AD) and eczema has become a prevalent issue for healthcare systems, especially in Western countries (1, 2). Meta-analyses estimated an increase in prevalence of FA even in adults, ranging from 3.5 to 35%. However, literature about FA in older people is still scarce and based on self-reported symptoms (3–6). The reasons for the increase in allergic sensitization, allergic reactions, as well as allergy severity, is multifactorial with environmental, epigenetic and genetic factors all playing a role in the origin and development of the disease (7). As a response, healthcare systems are now focused on a better understanding of the various modifiable eliciting factors of allergic diseases, which may then serve as targets for allergies primary prevention (8). Modifications in eating habits and nutrient intakes, which have accompanied the rise of modern societies, are well-recognized influencing factors on the growing prevalence of non-communicable diseases, including allergies. Indeed, nutrition may represent a key factor contributing to health throughout the life-course, as it is a fundamental cofactor for the development and maintenance of body composition and body functions (9, 10). In the case of allergies, many nutritional factors and dietary interventions have been studied for their function in promoting immune system maturation and immune tolerance to allergens. Among all, vitamins (A, C, D, and E), omega-3 long chain polyunsaturated fatty acids (n-3 LC-PUFA), copper, zinc, selenium, prebiotics, and probiotics have all sparked interest for their immunomodulatory functions, potentially promoting allergy prevention (11). Most recently, the concept that diet diversity (i.e., the variety of food being eaten) may itself influence allergic outcomes has also been proposed (12). Accordingly, the promotion of a more diverse diet may exert its influence on allergic outcome either directly, through the promotion of a healthy gut microbiome, and indirectly, with the introduction of potentially immunomodulatory nutrients (12, 13).

## Food Allergy

### Infants and Children

In infants and children, a preeminent role among allergic diseases is acted by food allergy (FA) which can occur as early as during the first year of life. Historically, this has led to the hypothesis that the exposition of potentially highly allergenic foods, such as hen's egg, peanuts and cow's milk should be avoided until the end of the first year of life. The basis of such recommendation was the belief that a certain degree of mucosal immunologic immaturity, present in infants, could negatively influence the challenge of the local immune cells with food antigens, ultimately resulting in increase of food allergen sensitization (14). However, this “late introduction” strategy has been weakened by the epidemiological evidence that tolerance to food allergens may more favorably be modulated by an early exposure to food allergens, even at 4–6 months of age. Later exposure, outside this “critical early window” may otherwise lead to oral tolerance failure, rising the risk of FA, coeliac disease, as well as autoimmunity phenomena (15). Accordingly, new guidelines for FA prevention have been issued proposing an earlier introduction of foods with proteins carrying a higher allergenic potential during complementary feeding as a better option to prevent and/or contrast the development of FA in the youngest population. Newer recommendations advise against delaying the introduction of solid foods after 4–6 months of age, while contextually discouraging early exposure to food allergens before 4 months of age (16, 17). Notably, the new recommendations are valid for all, regardless of the *a priori* hereditary predisposition of developing allergic reactions, as for the case of children with atopic dermatitis (AD). Indeed, the European Academy of Allergy and Clinical Immunology (EAACI) specifically recommends the “introduction of complementary foods after the age of 4 months for all children irrespective of atopic heredity” (16). The effectiveness of this early introduction strategy has yet to be clarified, with a recent review arguing that a positive effect in reducing FA occurrence has only been shown for early introduction of peanuts in infant diets. The potential positive effect of an early introduction of allergenic foods other than peanuts should be further investigated with randomized controlled trials (RCT) (1). The involvement of primary care pediatric centers and family pediatricians in RCT studies investigating more systematically early food allergens exposure and development of FA would be of great value also to understand the mechanisms responsible for FA. Whilst an earlier introduction of allergenic proteins has still to be challenged with most “at risk” foods, the delay in their introduction seems to be definitely out of any positive advantage (14).

An interesting insight on the potential mechanism underlying early development of FA or, on opposite, food tolerance has been provided by the recent “Dual allergen exposure hypothesis,” which proposes that the immune system response to food allergens may differ depending on the allergens first site of exposure during the first year of life (intestinal mucosal = food tolerance; percutaneous exposure = allergic sensitization) (1). The hypothesis is based on the observation that infants with skin problem, like as AD or eczema, may experience early percutaneous exposure to food antigens. Such early exposure, bypassing intestinal tolerance, may elicit a T helper type 2 (Th2) dominant response, leading to development of allergic sensitization. Consequently, avoiding oral introduction of food allergens, while still having environmental exposure to them, might elicit the occurrence of FA through transcutaneous sensitization, rather than prevent it (1, 18). According to this hypothesis, an earlier introduction of allergenic proteins should be supported to prevent a first environmental incidental exposure through the skin.

Besides conditions genetically predisposing to allergies (as for the case of children with AD) the rise of FA in recent years has also been linked to environmental factors, which may affect food tolerance. Specifically, unhealthy lifestyles and diet may negatively influence the intestinal microbiome and immune responses, as well as lead to nutritional imbalances concerning vitamins, antioxidants, fibers and fatty acids dietary intakes (19). The resident gastrointestinal microbiota has a modulatory role on the human immune system and can potentially influence allergies' occurrence, including FA (20). Indeed, prenatal life and early infancy are crucial for microbiome colonization and immune tolerance promotion. A healthy colonization may positively influence the development and maturation of the immune system and immune tolerance, eventually protecting the individual from allergic reaction, once complementary feeding is introduced (8, 21, 22). On the contrary, an unfavorable gut microbiota composition (i.e., microbial dysbiosis, commensal bacteria disruption, diversity reduction) has been associated with an increased risk of immune system dysregulation and allergies development (1, 23–25). To ameliorate the gut microbiota health in the perspective to prevent/manage some age relate conditions including FA, supplementations with prebiotics (i.e., Bifidobacterium breve M-16V) and probiotics (i.e., Lactobacillus rhamnosus GG) are being considered as promising strategies with potential implications in the prevention/management of FA, despite evidence on efficacy, particularly at long term, is still limited (26).

### Adults and Older People

Food allergies are usually regarded as a pediatric problem. However, it is expected that FA will be encountered more often even in older people given the increase in prevalence seen in the last decades (27). Unfortunately, literature about food allergies in older people (i.e., people aged 60 and older) is very scarce, leading to the impression that food allergies cannot be present and/or arise later in life (28). Nevertheless, FA may both persist in old age as well as they may occur for the first time during the later stages of life (29). Several changes occurring with aging have been suggested to play a role in mediating FA. In particular, the physiological decline in many functions and comorbidities related to aging itself may contribute to mask symptoms of FA (27, 30). To date, immune senescence has been evoked as one of the mechanisms underlying FA in older people. With aging, there is a decline in both innate and adaptive immune responses often associated to a pro-inflammatory profile (i.e., inflamm-aging) (31, 32). Indeed with aging a shift toward a pro-allergenic Th2 profile (27, 33, 34) may take place, at the point that a Th2 profile may not only be characteristic of childhood, but also be present in mid-to-later life (35, 36) therefore influencing the development of FA in older people (28). Even if an oral tolerance established during early life seems to be retained in older life, the ingestion of new dietary proteins may lead to *de novo* sensitization in older people (27, 28, 37–39). Recently, Gupta et al. (40) reported an estimated prevalence of FA of 8.8% among U.S. adults aged 60 and older, with the 5.5% of them developing FA as adults. Interestingly, they reported that shellfish allergy prevalence is nearly the same among individuals aged 18–29 years (i.e., 2.8 %) and among people aged 60 and older (i.e., 2.6 %), indirectly confirming a high rate of persistence through the life-course of this food allergy. The authors also reported a prevalence of milk allergy of 1.9% in adults aged 60 and above, in line with the other age groups considered. Another contributing factor for FA in older people is represented by the progressive alteration of intestinal permeability (41). Indeed, chronic low-grade inflammation seen with aging can increase the permeability of the tight junctions (42) resulting in a dysregulation of the mechanism of tolerance (27, 43). Additionally, atrophic gastritis commonly seen with aging and determining a decreased digestive ability, may lead to the persistence of undigested proteins developing as potential allergens (44, 45). Atrophic gastritis can also develop from alcohol abuse or chronic ingestion of proton pump inhibitors or antacids, further enhancing gastrointestinal permeability (46–48). In fact, age related modifications in the gut microbiota have been suggested as major contributors in mediating FA in older people (27). In particular, the gut microbiota has repeatedly been evoked as a mediator of a wide variety of age-related changes, ranging from innate immunity and sarcopenia up to cognitive function and, more broadly, frailty in general (49). In addition to unhealthy diets, antibiotic therapies, and prolonged treatments with proton pump-inhibitors, which are frequently (and often improperly) prescribed to older people, and even self-prescribed out of any medical advice, are known to negatively affect gut microbiota composition. Nutritional deficiencies typical of older ages, such as micronutrient deficiencies - including low levels vitamin D, zinc and iron - may play an additional role in sensitization to food allergens in older people (43). These micronutrients modulate immune responses and have antioxidant properties (39). For instance, zinc deficiency results in a reduction of T helper type 1 (Th1) cytokines, with a net shift toward Th2 responses, potentially favoring the occurrence of FA in older people (50, 51). Also vitamin D deficiency may influence the development of allergic reactions. In its active form (i.e., calcitriol), vitamin D has immunomodulatory properties through influencing both innate and adaptive immunity. In fact, calcitriol through T cells and antigen presenting cells promotes tolerance by inhibiting inflammatory processes and promoting the maturation of regulatory T cell subpopulations (52). Finally, iron deficiency is associated to a reduced antibody response, mainly in the IgG4 subclass, that inhibits the activation of effector cells through incorporating allergens before it binds to the IgE (28).

## Consequences of Food Allergies and of the Resulting Dietary Restriction on Body Health

Food allergies and the consequent dietary restriction resulting from the avoidance of allergenic foods may have negative effects on either short- and long-term outcomes. The development of FA in early life may negatively impact growth and more generally body composition. Moreover, an unsupervised dietary restriction has been correlated to presence of eating disorders later in life (i.e., adolescence) (53). Therefore, basic anthropometric and body composition indexes should be measured routinely in allergic subjects, or at least in those subgroups followed up in Centers where these sophisticated resources are available and/or feasible, in order to anticipate possible growth deviations and/or reduce the risk of malnutrition and malnutrition related comorbidities in later life (54, 55). Likewise, bone health should be regularly monitored in children who have a restricted diet, particularly in milk and milk-derived products, due to an allergic sensitization/disease to make sure that peak bone mass is attained and to prevent the consequences of poor bone health (i.e., fractures and skeletal pain) in later life (56). Given that the mainstay of the management of FA is based on the dietary elimination of major food allergens, attention should be paid to the risk of nutritional deficiencies. Inadequate intakes of specific nutrients may also be exacerbated by the anxiety of avoiding even minimal amounts of offending allergens in industry-based food preparations (57). Since a FA developed during childhood may persist also in adult life, this may determine a lifetime cumulative exposure to nutritional deficiencies, negatively affecting the functional reserves of the individual. For instance, older people need more proteins to counteract the age-related muscle decline (58, 59) and the avoidance of allergenic foods which are a major source of proteins (i.e., milk, egg, fish) may contribute to an inadequate protein intake (57). FA may be potentially regarded also as determinants of the so-called “anorexia of aging.” In fact, physiological changes seen with the aging process like as gastrointestinal alterations, which are frequently seen in FA, are well-recognized contributing factors to the anorexia of aging, thus augmenting the risk of malnutrition, frailty and sarcopenia (60, 61). Lastly, it is well-established that the exclusion of individual food categories from the diet may expose to other micronutrient deficiencies (i.e., calcium, selenium, iron, zinc, vitamin D, B vitamins). Ensuring an appropriate dietary quality, as well as adequate intakes of all macro- and micronutrients is therefore of pivotal importance in the management of FA. Indeed, as pointed out just recently by the “EAACI position paper on diet diversity in pregnancy, infancy and childhood,” studies from both developed and developing countries have shown that an increase in diet diversity is linked with an increase in nutrient adequacy (62). Under these premises, the importance of diet diversity may emerge as a novel strategy not only to prevent but also to manage FA throughout the life-course (57).

## Diet Diversity

The term “diet diversity” comprises two possible definitions (1) the variety of food being eaten or, (2) the number of different foods or groups of foods eaten over a reference period of time. Over the last decades, it has been observed that there is a positive association between diet diversity and numerous health outcomes. Most recently the same positive association has been investigated even between diet diversity and the occurrence of allergic diseases, including FA (12). The concept that diet diversity may have a positive influence on FA outcomes mainly derives from the observation that the promotion of diet diversity (and early food antigen introduction) in the first year of life is negatively associated with the occurrence of allergies, including FA. In particular, the relation of FA with diet diversity has been investigated in few recent studies so far. Indeed, regarding this lack of studies investigating the relationship between diet diversity and FA outcome, EAACI experts have claimed that there is a “true deficiency in the literature” (62). A negative association between increased diverse food intake in the first year of life and FA outcomes up to 6 years of age was first reported by Roduit et al. in 2014 (63). In the same year, in a case-control study, Grimshaw et al. observed that, compared to children diagnosed with FA by the age of 2 years, control children had a healthier diet in the first year of life, characterized by a higher introduction of fruit, vegetables, and home-made food (64). Most recently, Venter et al. (65) have examined four different (but internationally recognized) measurements' methods to define diet diversity and found that, regardless of the method applied, FA outcomes over the first 10 years of life were always reduced in children with higher diet diversity scores in the first year of life. Additional information on the importance of diet diversity in the prevention of FA derives from a paper by Nicklaus et al., who reported that increased diversity of cheese consumed at 18 months might have a protective effect on the development of FA (as well as AD) by the age of 6 years (66). An overview of the studies is presented in Table 1. Altogether, the studies here examined may suggest preliminary evidence that diet diversity should be promoted from very young ages, as early as with the beginning of complementary feeding. Diet diversity is also associated with a more favorable gut microbiome, so reinforcing the concept of the promising role of gut microbiota manipulation (either directly or indirectly) in inducing immune tolerance (67). Not only gut microbiome is responsible for the maintenance of the intestinal mucosa integrity, but it also explicates a regulatory action on the immune system. Therefore, diet diversity may promote allergy prevention and food tolerance by favoring the diversity of gut microbiome (12, 68). Diet diversity should be encouraged even after the “critical window” of the first year of life, in which diet diversity is believed to exert the most of its immunomodulatory effects, for the potential ability to modulate the tolerance to food allergens through the gut microbiome and its potential efficacy throughout the life-course. Lastly, diet diversity may additionally promote food tolerance favoring the introduction of immune-modulatory nutrients, such as omega-3 LC-PUFA and fibers, as well as vitamins and other substances with antioxidant properties, that are mostly represented in a diet funded on “diverse” foods (67). Given that a more diverse diet is associated with a healthier diet, regardless of age, the promotion of diet diversity should therefore be supported in both children and adults to prevent FA on one hand, and as much as possible in those subjects with FA still following food restricted diets. Within this perspective, new dieto-therapeutic approaches should be challenged in clinical trials in the forthcoming years, at all ages, through the life span.

**Table 1:** Overview of the original studies exploring the effects of diet diversity for the prevention of food allergy.

**References**	**Study design**	**Aims**	**Relevant results**
**Original studies**			
Roduit et al. (63)	Birth cohort study	To investigate the association between the introduction of food during the first year of life and the development of asthma, allergic rhinitis, FA, or atopic sensitization.	Increased diverse food intake in the first year of life is negatively associated with the occurrence of FA outcomes in children up to 6 years of life
Grimshaw et al. (64)	Case-control study within a birth cohort study	To present data on the incidence of FA in the UK within a general UK cohort and to investigate the possible risk factors for IgE and non-IgE mediated FA.	Compared to children diagnosed with FA by the age of 2 years, control children had a healthier diet in the first year of life, characterized by a higher introduction of fruit, vegetables, and home-made food
Venter et al. (65)	Birth cohort study	To investigate the association between 4 different measures of DD during infancy and development of FA over the first decade of life.	Regardless of the method applied to measure DD, FA outcomes over the first 10 years of life were always reduced in children with higher DD scores in the first year of life.
Nicklaus et al. (66)	Birth cohort study	To evaluate whether cheese consumption is associated with allergic diseases, like as FA.	Increased diversity of cheese consumed at 18 months might have a protective effect on the development of FA by the age of 6 years

*DD, Diet Diversity; FA, Food Allergy; UK, United Kingdom*.

## Concluding Remarks

In the last decades, the rise in allergic diseases, including FA, is becoming a prevalent issue for healthcare systems. Contrary to what is believed, FA are not only an issue mainly related to pediatric ages, as they can also persist or even occur for the first time in adult life. Although the mechanisms involved may differ at the two extremes of life, FA may be more difficult to identify in older people who are characterized by a high clinical complexity with multiple chronic comorbidities and mutually interactive syndromes. Indeed, FA in older life may be observed as a corollary of the aging process which is characterized by mechanisms like as inflamm-aging, immunosenescence and gut microbiota alterations. Diet diversity is emerging as a new strategy to prevent the occurrence of FA in early life with potential implications in later stages of the life too. Additionally, gut microbiota manipulation represents a promising approach in preventing and managing FA despite further evidence is needed.

## Author Contributions

DA and GS equally contributed to conceptualizing and writing the manuscript. CA and MC edited and revised the manuscript. All authors approved the final version of the manuscript.

## Funding

This study was supported by a contribution from the Italian Ministry of Health (Istituto di Ricovero e Cura a Carattere Scientifico- IRCCS grant).

## Conflict of Interest

The authors declare that the research was conducted in the absence of any commercial or financial relationships that could be construed as a potential conflict of interest. The handling Editor declared a past co-authorship with one of the authors CA.

## Publisher's Note

All claims expressed in this article are solely those of the authors and do not necessarily represent those of their affiliated organizations, or those of the publisher, the editors and the reviewers. Any product that may be evaluated in this article, or claim that may be made by its manufacturer, is not guaranteed or endorsed by the publisher.
